# An Atypical Presentation of Twiddler’s Syndrome: A Case Report

**DOI:** 10.7759/cureus.27207

**Published:** 2022-07-24

**Authors:** Hugo Liano Calavia, Menna Awadalla, Syed Alishan Nasir, Jean Hammel

**Affiliations:** 1 Internal Medicine, Norwalk Hospital, Norwalk, USA; 2 Internal Medicine, University of Vermont, Burlington, USA; 3 Emergency Medicine, Norwalk Hospital, Norwalk, USA

**Keywords:** hyperventilation, alkalosis, twiddler's syndrome, cardiology devices, implantable-cardioverter defibrillator

## Abstract

The prevalence of implanted pacemaker/defibrillator devices continues to rise. Automatic implantable cardioverter-defibrillator (AICD) lead displacement (Twiddler’s syndrome) is an uncommon form of the device malfunction, usually presenting with cardiac symptoms. We present a case of Twiddler’s syndrome with an atypical presentation, accompanied by critical alkalosis on arterial blood gas. Considering Twiddler’s syndrome as part of the differential diagnosis in patients with implanted devices and utilizing remote ICD interrogation may improve the care of patients presenting with device malfunction.

## Introduction

Approximately 3 million people in the United States live with implanted pacemaker/defibrillator devices. An average of 600,000 new devices are implanted each year, and the incidence continues to rise [1, 2]. Although not exceedingly often, pacemaker malfunction does occur. Common presentations of device malfunction include inappropriate defibrillation, chest pain, shortness of breath, palpitations, and dizziness. Approximately 13% of the time, however, patients can present with non-cardiac symptoms [3].

## Case presentation

A 33-year-old male with a past medical history of nonischemic cardiomyopathy with an ejection fraction 15-20%, status post bi-ventricular automated implantable cardiac defibrillator (AICD) placement, gastroesophageal reflux disease (GERD), hyperthyroidism, type II diabetes mellitus, hypertension, and asthma was brought to the ED by ambulance with a chief complaint of palpitations and upper abdominal pain. Upon arrival, the patient reported a seven-day history of acute, worsening, severe, intermittent abdominal and substernal pain accompanied by a “twitching” sensation in his upper abdomen, an “irritated diaphragm”, palpitations, shortness of breath, hiccups, nausea, decreased appetite, dry cough, lightheadedness, and “reflux sensation”. Five days earlier, the patient had presented to the ED at a different hospital with similar symptoms, primarily emphasizing right upper quadrant abdominal pain. The patient was discharged with a presumptive diagnosis of musculoskeletal pain and was prescribed Percocet. However, the patient’s symptoms continued to worsen, prompting his visit to our ED.

Pertinent home medications included metoprolol succinate, sacubitril-valsartan, ivabradine, aspirin (81 mg), torsemide, spironolactone, metformin, empagliflozin, methimazole, and esomeprazole. The patient reported compliance with all medications. Upon arrival, the patient was tachypneic (respiratory rate 24-28) but saturating greater than 95% on room air. The remaining vital signs were within normal limits. The patient was somnolent but easily arousable. He intermittently seemed to be jostled out of his sleep as if being defibrillated, although no defibrillator spike was noted on the cardiac monitor. Rhythmic twitching of his left upper extremity was noted. He had no reproducible tenderness to palpation of the abdomen. A regular rate and rhythm were appreciated on the cardiac exam with no murmurs, rubs, or gallops. No peripheral edema was noted. The remainder of the physical exam was unremarkable.

Chemistries were unremarkable. A mild leukocytosis of 10.6 x 109/L was noted. Urine toxicology screen, serum salicylate, and serum thyroid-stimulating hormone (TSH) levels were all within normal limits, and troponin was undetectable.

**Figure 1:**
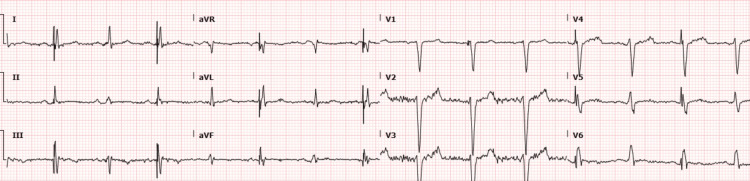
ECG showing biventricular-paced normal sinus rhythm.

Given the patient’s somnolence and body habitus, an arterial blood gas was ordered to assess for possible hypercarbia. Surprisingly, it displayed a marked mixed respiratory and metabolic alkalosis with a pH of 7.66 (7.35-7.45), pCO_2_ of 23.8 mmHg (35-45 mmHg), pO_2_ of 129 mmHg (75-100 mmHg) and HCO_3_ 31.6 mEq/L (22-29 mEq/L). A repeat draw was obtained for verification, noting an even higher pH of 7.70. An ECG (Figure 1) displayed a biventricular-paced normal sinus rhythm. Five weeks prior, the patient’s AICD had been upgraded from single-chamber to a bi-ventricular model, and an atrial lead was also placed at that time.

A representative from the AICD’s manufacturer, who had previously been contacted, arrived at the bedside. He interrogated the device and explained that the atrial lead appeared to have dislodged and migrated to a different site. This rendered the device unable to sense intrinsic sinoatrial impulses, and it was constantly pacing within its new location. In addition, the interrogation revealed that no defibrillator shocks had been delivered. As a result, the patient’s atrial lead was disabled, switching mode to ventricular-only pacing, with a near-instantaneous resolution of the patient’s abdominal symptoms.

The patient was admitted to ICU for management of his acid-base derangements. He only received three runs of 10 mEq IV potassium chloride during this time. Preparations for hemodialysis were made in the event of worsening alkalosis. His home diuretics (torsemide, spironolactone) were held. A repeat arterial blood gas was obtained, which showed a pH of 7.50, pCO_2_ 41.1 mmHg, PO_2_ 87 mmHg, and HCO_3_ 31.6mEq/L, significant for resolving alkalosis. Over the course of his hospital stay, his tachypnea and somnolence resolved, leukocytosis corrected, and he remained stable and asymptomatic. The patient was discharged the following day with plans to see his electrophysiologist for AICD lead revision, and his acid-base disorder was attributed to hyperventilation in the setting of lead displacement.

## Discussion

While the differential for our patient’s initial presentation was broad, AICD dysfunction (as previously discussed) appeared to be the most likely diagnosis, given the near-instantaneous resolution of symptoms following deactivation of the atrial lead and the fact that no other unifying diagnosis was identified.

This presentation is consistent with Twiddler’s syndrome, a disorder estimated to occur in 0.07-7% of patients with an AICD or permanent pacemaker, with most cases occurring within a year of implantation [4]. In our patient, his single chamber AICD was recently upgraded to a biventricular AICD on September 1, 2020, five weeks prior to this presentation. This syndrome occurs secondary to external manipulation of the device by the patient, resulting in the spinning of the pulse generator and, subsequently, lead dislodgement [5]. Commonly reported symptoms include chest pain [6, 7], shock-like sensations/contractions in the chest [6, 8], and syncope [4] - all of which are relatively easy to reconcile with a cardiac etiology. However, diaphragmatic pacing with accompanying abdominal pulsation (secondary to ipsilateral phrenic nerve stimulation) and upper extremity twitching (secondary to pacing of the brachial plexus) have also been reported in the literature and were present in our patient [5].

Our patient’s intractable hiccups were likely driven by this direct diaphragmatic irritation, and paradoxically led to his unusual somnolence on ED presentation, as the hiccups and repeated stimulation through inappropriate pacing had kept him awake for most of the past few nights.

The differential for metabolic alkalosis is also broad, including renal causes (hydrogen and potassium wasting), GI (chloride losses secondary to vomiting or nasogastric suctioning), and hypovolemia (contraction alkalosis), amongst others [9]. Medication's adverse effects are often a culprit, with loop and thiazide diuretics common offenders [10]. Other offending agents would include antacids in combination with calcium supplements leading to the milk-alkali syndrome and magnesium oxide supplements leading to hypokalemia when taken in excess [11]. In our patient's case, the most likely etiology is an excess diuretic intake, as it came to our attention that he mistakenly was taking torsemide at a higher dose and frequency than prescribed.

Our patient's arterial blood gas is an interesting finding, with mixed alkalosis being an uncommon presentation. Alkalosis is, however, reported as a complication of pacemaker/AICD malfunction [12]. Primary respiratory alkalosis may be driven by any process impairing oxygenation and/or leading to tissue hypoxia, causing hyperventilation as a response. Non-hypoxic differentials include CNS insult (causing ventilatory dysregulation), pain, or anxiety. It is likely that our patient's hyperventilation was driven primarily by the pain, discomfort, and anxiety associated with the repeated delivery of pacemaker discharges irritating local tissue and direct diaphragmatic stimulation by the pacemaker, as discussed above.

## Conclusions

Twiddler's syndrome can present in many atypical ways, including abdominal pain, diaphragmatic irritation, and hyperventilation, as noted in our patient. Approximately 13% of the time, AICD malfunction can present with non-cardiac symptoms. For most patients with AICDs and any thoracic or abdominal complaint, an EKG and a chest X-ray in the ED should be performed, as these can quickly detect lead misplacement and pacer malfunction. Confirming the diagnosis through device interrogation is relatively easy, particularly given new remote options which involve an on-site device that interrogates the ICD and transmits the information to the device manufacturer to generate a report quickly. This way, the data can be promptly reviewed without waiting for a representative to arrive at the ED personally. 

In conclusion, keeping device malfunction in mind as a differential diagnosis in patients with implanted devices may prove beneficial to ED physicians and patients by improving diagnostic accuracy and delivery of appropriate care in a timely manner. Twiddler's syndrome is a rare cause of hyperventilation and, in our case, critical alkalosis.
